# Supplementary material for: Alcohol use among Congolese Babembe male refugees in Tarrant County: A qualitative study
Source: PLoS One. 2022 Aug 3;17(8):e0272293. doi: 10.1371/journal.pone.0272293 (PMC9348719; doi:10.1371/journal.pone.0272293)
Supplement: Attachment — Submitted filename: Response to Reviewers.docx [file pone.0272293.s001.docx]

**Response to Reviewers**

**Journal Requirements:**

When submitting your revision, we need you to address these additional requirements.

1. Please ensure that your manuscript meets PLOS ONE's style requirements, including those for file naming. The PLOS ONE style templates can be found at

[https://journals.plos.org/plosone/s/file?id=wjVg/PLOSOne_formatting_sample_main_body.pdf](https://nam04.safelinks.protection.outlook.com/?url=https%3A%2F%2Fjournals.plos.org%2Fplosone%2Fs%2Ffile%3Fid%3DwjVg%2FPLOSOne_formatting_sample_main_body.pdf&data=04%7C01%7CEvangeline.Baker%40unthsc.edu%7Cb45772a9179646142c1108d9d47c49ea%7C70de199207c6480fa318a1afcba03983%7C0%7C0%7C637774453626539024%7CUnknown%7CTWFpbGZsb3d8eyJWIjoiMC4wLjAwMDAiLCJQIjoiV2luMzIiLCJBTiI6Ik1haWwiLCJXVCI6Mn0%3D%7C3000&sdata=XAwCCUD5SvaXtjWNyupBzULUnR4GVc3trNPqzfEkZ88%3D&reserved=0)  and

[https://journals.plos.org/plosone/s/file?id=ba62/PLOSOne_formatting_sample_title_authors_affiliations.pdf](https://nam04.safelinks.protection.outlook.com/?url=https%3A%2F%2Fjournals.plos.org%2Fplosone%2Fs%2Ffile%3Fid%3Dba62%2FPLOSOne_formatting_sample_title_authors_affiliations.pdf&data=04%7C01%7CEvangeline.Baker%40unthsc.edu%7Cb45772a9179646142c1108d9d47c49ea%7C70de199207c6480fa318a1afcba03983%7C0%7C0%7C637774453626539024%7CUnknown%7CTWFpbGZsb3d8eyJWIjoiMC4wLjAwMDAiLCJQIjoiV2luMzIiLCJBTiI6Ik1haWwiLCJXVCI6Mn0%3D%7C3000&sdata=346BOZNNBEjvldSXgqzvSdl85wlCKtP3kVC9eHWe28Q%3D&reserved=0)

We have updated our format to reflect the changes needed to meet journal requirements.

2. We note that the grant information you provided in the ‘Funding Information’ and ‘Financial Disclosure’ sections do not match.

When you resubmit, please ensure that you provide the correct grant numbers for the awards you received for your study in the ‘Funding Information’ section.

We will update it to match funding information. Thank you.

3. Thank you for stating the following financial disclosure:

“No. The funders had no role in study design, data collection and analysis, decision to publish, or preparation of the manuscript”

At this time, please address the following queries:

a)        Please clarify the sources of funding (financial or material support) for your study. List the grants or organizations that supported your study, including funding received from your institution.

Updated.

b)        State what role the funders took in the study. If the funders had no role in your study, please state: “The funders had no role in study design, data collection and analysis, decision to publish, or preparation of the manuscript.”

Updated in manuscript.

c)        If any authors received a salary from any of your funders, please state which authors and which funders.

d)        If you did not receive any funding for this study, please state: “The authors received no specific funding for this work.”

Updated.

Please include your amended statements within your cover letter; we will change the online submission form on your behalf.

4. In your Data Availability statement, you have not specified where the minimal data set underlying the results described in your manuscript can be found. PLOS defines a study's minimal data set as the underlying data used to reach the conclusions drawn in the manuscript and any additional data required to replicate the reported study findings in their entirety. All PLOS journals require that the minimal data set be made fully available. For more information about our data policy, please see [http://journals.plos.org/plosone/s/data-availability](https://nam04.safelinks.protection.outlook.com/?url=http%3A%2F%2Fjournals.plos.org%2Fplosone%2Fs%2Fdata-availability&data=04%7C01%7CEvangeline.Baker%40unthsc.edu%7Cb45772a9179646142c1108d9d47c49ea%7C70de199207c6480fa318a1afcba03983%7C0%7C0%7C637774453626539024%7CUnknown%7CTWFpbGZsb3d8eyJWIjoiMC4wLjAwMDAiLCJQIjoiV2luMzIiLCJBTiI6Ik1haWwiLCJXVCI6Mn0%3D%7C3000&sdata=QWax8y6ylOOQD%2BQXW7stDPjr39z1FxYoCHCmYDHhRrU%3D&reserved=0).

Upon re-submitting your revised manuscript, please upload your study’s minimal underlying data set as either Supporting Information files or to a stable, public repository and include the relevant URLs, DOIs, or accession numbers within your revised cover letter. For a list of acceptable repositories, please see http://journals.plos.org/plosone/s/data-availability#loc-recommended-repositories. Any potentially identifying patient information must be fully anonymized.

Important: If there are ethical or legal restrictions to sharing your data publicly, please explain these restrictions in detail. Please see our guidelines for more information on what we consider unacceptable restrictions to publicly sharing data: [http://journals.plos.org/plosone/s/data-availability#loc-unacceptable-data-access-restrictions](https://nam04.safelinks.protection.outlook.com/?url=http%3A%2F%2Fjournals.plos.org%2Fplosone%2Fs%2Fdata-availability%23loc-unacceptable-data-access-restrictions&data=04%7C01%7CEvangeline.Baker%40unthsc.edu%7Cb45772a9179646142c1108d9d47c49ea%7C70de199207c6480fa318a1afcba03983%7C0%7C0%7C637774453626539024%7CUnknown%7CTWFpbGZsb3d8eyJWIjoiMC4wLjAwMDAiLCJQIjoiV2luMzIiLCJBTiI6Ik1haWwiLCJXVCI6Mn0%3D%7C3000&sdata=CNzi%2BhdjkUaNyhaO%2F9y1Q7EzpZtx3PrSTGucGzCJCis%3D&reserved=0). Note that it is not acceptable for the authors to be the sole named individuals responsible for ensuring data access.

We will update your Data Availability statement to reflect the information you provide in your cover letter.

Data cannot be shared publicly because of limitations of the participant consents approved by the North Texas Regional Institutional Review Board. The de-identified minimal data set will be available if requested from Dr. Brian A Gladue, Vice President for Research, Institutional Official, Director of the North Texas Regional IRB [brian.gladue@unthsc.edu](mailto:brian.gladue@unthsc.edu)

5. We note that you have stated that you will provide repository information for your data at acceptance. Should your manuscript be accepted for publication, we will hold it until you provide the relevant accession numbers or DOIs necessary to access your data. If you wish to make changes to your Data Availability statement, please describe these changes in your cover letter and we will update your Data Availability statement to reflect the information you provide.

[Note: HTML markup is below. Please do not edit.]

Reviewers' comments:

Reviewer's Responses to Questions

**Comments to the Author**

1. Is the manuscript technically sound, and do the data support the conclusions?

The manuscript must describe a technically sound piece of scientific research with data that supports the conclusions. Experiments must have been conducted rigorously, with appropriate controls, replication, and sample sizes. The conclusions must be drawn appropriately based on the data presented.

Reviewer #1: Yes

Reviewer #2: Partly

2. Has the statistical analysis been performed appropriately and rigorously?

Reviewer #1: N/A

Reviewer #2: N/A

3. Have the authors made all data underlying the findings in their manuscript fully available?

The [PLOS Data policy](https://nam04.safelinks.protection.outlook.com/?url=http%3A%2F%2Fwww.plosone.org%2Fstatic%2Fpolicies.action%23sharing&data=04%7C01%7CEvangeline.Baker%40unthsc.edu%7Cb45772a9179646142c1108d9d47c49ea%7C70de199207c6480fa318a1afcba03983%7C0%7C0%7C637774453626539024%7CUnknown%7CTWFpbGZsb3d8eyJWIjoiMC4wLjAwMDAiLCJQIjoiV2luMzIiLCJBTiI6Ik1haWwiLCJXVCI6Mn0%3D%7C3000&sdata=ETLc3NgHHYcdqYS5jAbB2u6tMKjGYnTWgskRZPfl3Sg%3D&reserved=0) requires authors to make all data underlying the findings described in their manuscript fully available without restriction, with rare exception (please refer to the Data Availability Statement in the manuscript PDF file). The data should be provided as part of the manuscript or its supporting information, or deposited to a public repository. For example, in addition to summary statistics, the data points behind means, medians and variance measures should be available. If there are restrictions on publicly sharing data—e.g. participant privacy or use of data from a third party—those must be specified.

Reviewer #1: Yes

Reviewer #2: Yes

4. Is the manuscript presented in an intelligible fashion and written in standard English?

PLOS ONE does not copyedit accepted manuscripts, so the language in submitted articles must be clear, correct, and unambiguous. Any typographical or grammatical errors should be corrected at revision, so please note any specific errors here.

Reviewer #1: Yes

Reviewer #2: Yes

5. Review Comments to the Author

Please use the space provided to explain your answers to the questions above. You may also include additional comments for the author, including concerns about dual publication, research ethics, or publication ethics. (Please upload your review as an attachment if it exceeds 20,000 characters)

Reviewer #1: The purpose of this study is to explore the determinants and practises that contribute to alcohol use among one of the world's most vulnerable populations. While this work has major implications for public health, it should clarify or address the following points.
The title of the article is 'Alcohol usage among Congolese Babembe refugees in Tarrant County: A qualitative study.' Kindly include among male refugees, as this study is mostly focused on male participation.

Thank you, it has been updated.

Abstract: Indicate in approach which approach you used, such as phenomenology, ethnography, or narrative. Similarly, make reference to the analysis method of 'thematic content analysis.'

Thank you, it has been updated.

Introduction: There are no comments.

Methods:
It stated unequivocally that 19 individuals were chosen using snow-ball sampling. How many focus groups were held by the authors? Each FGD recorded the number of participants. I believe that facilitating 19 participants in a single FGD is tough. How the author dealt with it. All of these approaches put limits on your data collection procedures. Alternatively, the author may refer to 19 interviews conducted with semi-structured open-ended questions.

Thank you, it has been updated with in the manuscript.

How many individuals did you approach? And how many are unwilling to participate, and what are their reasons? Are there any additional non-participants present during the FGDs?

Thank you, we have updated it in the manuscript, and there were no others present in the focus group than those participating.

Kindly include one sentence describing each author's educational background and professional experience in qualitative research, as well as how their multidisciplinary backgrounds help to extend the scope of the findings' interpretation. You may mention investigator triangulation here.

We have updated it to include authors backgrounds. Thank you.

Results:
The participants characteristics you can move to methods section. Since it is only 19 participants instead of table you may summarize the key participants characteristics.
In each quotation mention the age and years lived in refugees camp of the participant.

Because of the sensitivity of the community to confidentiality, as well as the small number of people within this specific vulnerable population, adding identifiers to comments would be highly likely for community members to be identified. According to IRB requirements we would not be able to add this to the manuscript.

In Subtheme 2: Alcohol abuse in refugee camps. Reduce the quotation length and describe the content in detail.

Updated, thank you.

The ‘Subtheme 5: America has destroyed our culture, and Subtheme 6: Loss of traditional ways to control alcohol abuse’ may be combined.

Thank you, updated.

Similarly, sub-theme 8 to 11 may be classified as two broad sub-themes.

Updated, thank you.

There is need of table on coding tree. Example of coding tree given below.
Themes
Sub-themes
Codes

We reduced the number of sub themes to 9. Thank you.

Discussion:
Mention one paragraph on ‘Implication for policy and practice’
Instead of limitations, put the heading as ‘Methodological considerations’ and discus both strength and limitations of the study.

Great suggestions. Thank you. We have updated it to include both of those points.

Conclusion section is missing.

Updated and included conclusion. Thank you

Reviewer #2: The central contribution of the manuscript is to help the reader understand the factors contributing to alcohol abuse among the Babembe refugees who are originally from Congo. While the empirical material and analysis indicate the relationship between the refugee status and alcohol abuse, it may require more nuanced analysis of the data to present the argument better. The connection between the theoretical argument and the empirical material needs to be strengthened

Thank you, we updated the information with this in mind.

Some specific comments:
1. The authors should include a justification of why FGD was conducted with 19 members in the methods section. While they discuss this in the limitations, it may help the reader to contextualise the results if it is mentioned upfront.

Thank you, this was updated in the methods section.

2. FGD using a semi-structured guide make better sense than mentioning ‘semi-structured FGD’.

Thank you, updated.

3. The analysis section should be strengthened by including a description of the analysis process and epistemological stance of the researchers in choosing thematic content analysis. They should also provide a reference for the analysis framework.

Thank you, updated.

4. Use of identifiers that do not reveal the identities of the respondents along with the quotations is important.

Because of the sensitivity of the community to confidentiality, as well as the small number of people within this specific vulnerable population, adding identifiers to comments would be highly likely for community members to be identified. According to IRB requirements we would not be able to add this to the manuscript.

5. For the subtheme 2, the quote on TKK does not seem to fit into the theme of alcohol abuse. The quote could be used to substantiate the existing efforts to tackle the alcohol abuse problem and the community participation.

Thank you. We have updated it to be more fitting.

6. Subtheme 4 indicates ease of access and low cost of alcohol. The theme can be rephrased to effectively communicate to the reader

Thank you, we have updated it.

7. Under theme 2, all the subthemes are overlapping. There has to be more nuanced presentation of the data in this theme.

Thank you, we have updated it.

8. Are the themes and subthemes based on the verbatim from the FGD?

We included themes commonly heard in the focus group, but not all terms were exact quotes used by participants.

9. Theme 3 and the sub themes need to be rephrased.

Updated, thank you.

10. Sub-theme 10 is misleading about empowerment. The statement and the quote convey that beating wife is empowerment for men. I’m sure that that the community and the researchers do not subscribe to this!!

Thank you for pointing that out. We have updated it.

11. The discussion could be have been more effective if discussion around larger social determinants, issues around racism shape the experience of the refuges in US.

We would love to build on this paper to include the larger social determinants in the next manuscript. However, the primary theme the community members wanted to express was their loss of cultural identity because of forced migration. We hope to publish further studies with this included.

12. The manuscript should have included some discussion around how these factors were dealt with among other refugees or could be dealt with through appropriate interventions among the Babembe.

Thank you for your feedback. We have included the further research and the desired policy and procedure changes for the refugee communities.
